# Splicing Hollow-Core Fiber with Standard Glass-Core Fiber with Ultralow Back-Reflection and Low Coupling Loss

**DOI:** 10.1021/acsphotonics.4c00677

**Published:** 2024-07-29

**Authors:** Bo Shi, Cong Zhang, Thomas Kelly, Xuhao Wei, Meng Ding, Meng Huang, Songnian Fu, Francesco Poletti, Radan Slavík

**Affiliations:** † Optoelectronics Research Centre, University of Southampton, Southampton SO17 1BJ, U.K.; ‡ School of Information Engineering, Guangdong University of Technology, Guangzhou 510006, China

**Keywords:** fiber optics, optical fiber, connection of hollow-core optical fibers, mode matching, Fresnel back-reflection

## Abstract

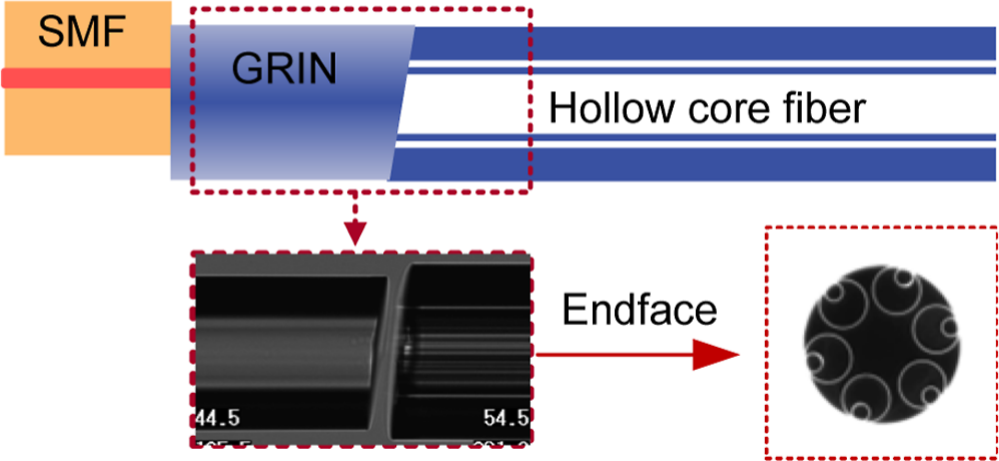

A main, yet-unsolved challenge in splicing hollow-core fiber (HCF) into standard single-mode fiber (SMF) systems lies in managing the strong Fresnel back-reflection that occurs when the light travels from the empty core of the HCF into the glass core of the SMF or vice versa. This impacts the performance of fiber systems that combine SMFs and HCFs due to effects such as multipath interference. Here, we demonstrate a new technique that combines angle-cleaving the HCF, which reduces the back-reflection, with offset-splicing the mode-field adapter to the SMF, which compensates for the refraction at the glass–air interface, enabling us to achieve low coupling loss. We first analyze this novel configuration via simulations and show that it is possible to achieve a coupling loss that is comparable to a conventional flat-cleaved splice. Subsequently, we fabricate an SMF–HCF connection with a loss of 0.6 dB prior to arcing (1.2 dB after splicing) and ultralow back-reflection (−64 dB) by applying an optimized 4.5° angle and 5 μm offset. To the best of our knowledge, this is the first low-insertion-loss spliced SMF–HCF connection where a widely acceptable level of back-reflection of <−60 dB is achieved.

## Introduction

Hollow-core fiber (HCF) guides light through an empty core, bringing many advantages compared to traditional fibers, such as single-mode optical fibers (SMFs), where light propagates through solid glass material. These advantages include lower contribution from the glass absorption and scattering, enabling HCFs to have low attenuation^1^ even at wavelengths where glass-core fibers are relatively lossy. It ranges from the visible wavelengths^2,3^ of interest to quantum technologies, e.g., used for transmitting quantum states in quantum communication,^4^ through the 1000 nm spectral region relevant for high-power lasers, all the way to mid-infrared^5^ up to the 5 μm region, e.g., HCF has been employed as a gas cell for nitrous oxide detection at 5.36 μm,^6^ enabling transmission at wavelengths relevant for applications in sensing.^7−9^ Many of these applications can further benefit from other properties of HCF, e.g., it can transmit kilowatt laser powers^10^ over kilometer distances^11^ due to low nonlinearity. In gas photonics, the effective overlap between the light beam and the gas inside the HCF is beneficial for gas lasers and sensing.^9,12−14^ Another example is HCFs’ low phase and delay sensitivity to temperature, making them superior for temperature-insensitive fiber interferometers and of interest in applications such as metrology.^15,16^

However, integrating HCF into existing SMF-based systems brings challenges. One is the connection loss due to the mode field size mismatch between the HCF and SMF. Typically, the fundamental mode of low-loss HCFs has a mode field diameter (MFD) about two times larger than SMF at 1550 nm, and this mismatch can be even bigger at other wavelengths. Several methods have been proposed to provide necessary MFD adaptation, for example, SMF tapering,^17^ inverse taper,^18^ thermally expanded core,^19^ or using a short segment of gradient index fiber (GRIN) close to 1/4 pitch length inserted between SMF and HCF.^20^ All of these methods have the potential to perfectly adapt the MFD of the SMF and HCF fundamental modes, resulting in the coupling loss being limited by the mode field shape mismatch (e.g., around 0.1 dB^21^) and the Fresnel reflection that for the silica glass–air interface is about 0.15 dB (corresponding to 3.5%). Coupling limited by the mode field shape mismatch has already been demonstrated using the GRIN mode field adaptation method in conjunction with an antireflective coating (AR), achieving a coupling loss of 0.08 dB.^20^ This low-loss connection was then secured via gluing, as the AR coating was reported to deteriorate when fusion splicing solid-core fiber with HCF.^22^ However, fusion splicing has been established as a preferred method in connecting SMFs, providing a chemically stable connection that performs over large temperature ranges and has been proven to remain stable over very long periods of time.

**Figure 1:**
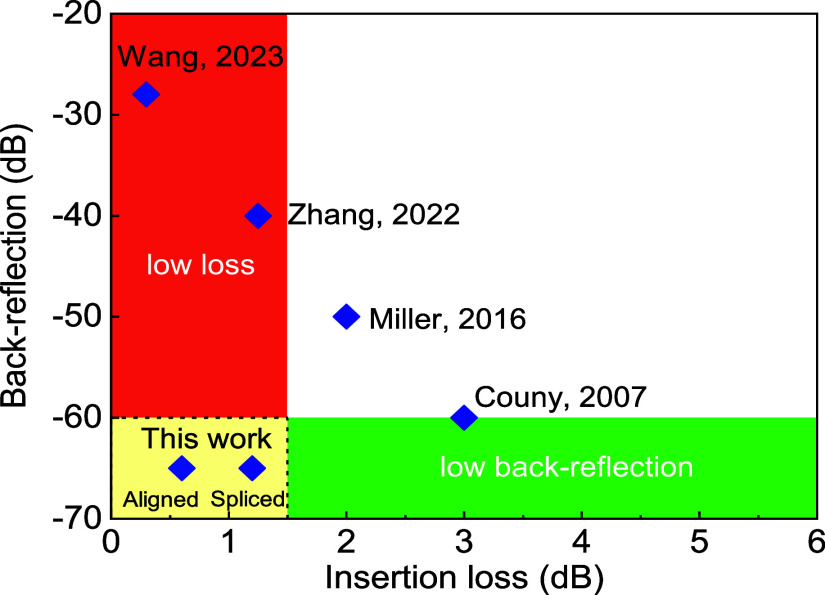
Back-reflection and coupling loss of spliced SMF–HCF connection.

**Figure 2:**
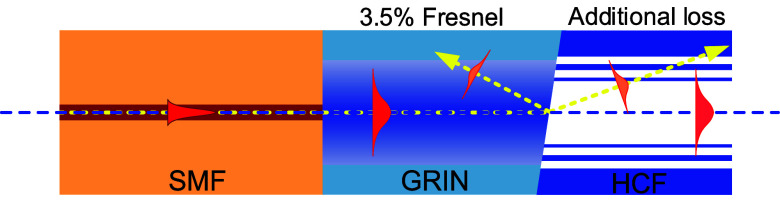
Schematics of SMF–HCF coupling with GRIN mode field adapter that is angle-cleaved to reduce the back-reflection. The angle cleave, however, also increases coupling loss into the fundamental HCF mode due to the refraction at the GRIN–HCF interface.

Unfortunately, all research into low-loss and low-back-reflection HCF–SMF fusion splicing has shown a trade-off between connection loss and back-reflection, as summarized in Figure 1. Wang et al. spliced an AR coated flat-cleaved thermally expanded fiber, achieving a low coupling loss of 0.2 dB; however, the AR coating deteriorated during fusion splicing, providing only a modest level of back-reflection suppression of −28 dB.^22^ Miller et al. and Couny et al. spliced angle-cleaved SMF to HCF, achieving a low back-reflection level of −60 dB at the expense of a degraded coupling loss.^23,24^ This trade-off is explained in Figure 2 using GRIN as the mode field adapter; however, the principle of this trade-off is similar in all published splicing methods, which reduce the back-reflection via angle-cleaving. When the MFD-adapted mode reaches the angle-cleaved end face of the GRIN, Fresnel-reflected light will not be coupled back into the SMF thanks to the angle-cleaving, enabling low back-reflection. However, the transmitted light will refract at the angled end facet, meaning that the output beam exits the fiber at an angle with respect to the HCF axis, causing reduced coupling into the fundamental mode with part of the energy coupled into higher order modes (HOMs). Larger cleave angles will reduce the back-reflection while increasing unwanted coupling into HOMs due to the increased refraction. Previously, we investigated this trade-off in detail, enabling us, for example, to achieve a moderate level of back-reflection of −40 dB with an acceptable level of coupling loss of 1.3 dB.^25^

Here, we suggest a method that resolves the above-mentioned trade-off, enabling for a spliced SMF–HCF connection that simultaneously has both low loss and low back-reflection. It uses a GRIN mode field adapter, to which the SMF is spliced, with an offset that compensates for the refraction at the angle-cleaved interface. Such a connection that achieves <−60 dB back-reflection with the coupling loss being potentially limited only by the mode field shape and Fresnel loss (about 0.25 dB together for current low loss HCFs^20^) is of interest for a multitude of applications in different areas. For example, the parasitic Fabry–Perot resonances in HCF-based gas cells used for absorption spectroscopy can be decreased without any AR coating.^26^ Reduction of these parasitic resonances also improves bit-error ratio in high-speed data transmission with HCF.^22,27^ In metrology, examples include HCF-based polarization-insensitive interferometry using Faraday rotator-assisted Michelson interferometers^28^ and HCF-based gyroscopes^29^ or high dynamic range HCF-based optical time domain reflectometers (OTDRs) for distributed fiber sensing.^30,31^

## Principle of Operation

**Figure 3:**
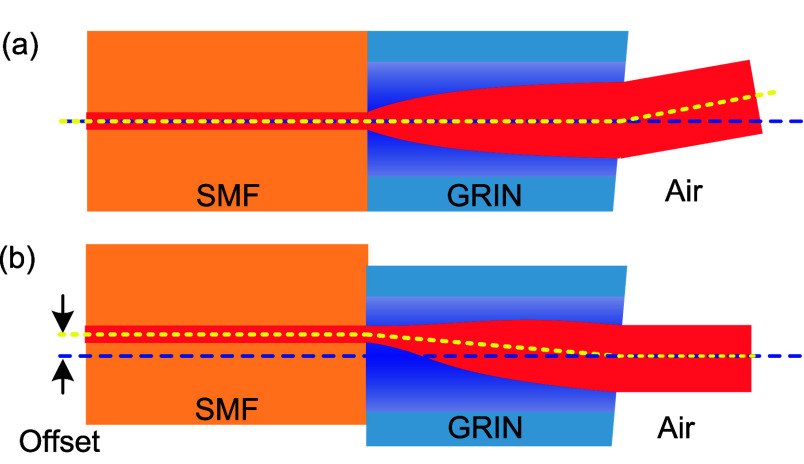
Light trace for SMF spliced with angle-cleaved GRIN without any offset, producing angle-propagating beam at the output (a) and its modification with optimized offset between the SMF and GRIN axes, producing output beam propagating along the optical axis (b).

The principle of operation is sketched in Figure 3. In a previously demonstrated configuration^25^ (Figure 3a), the beam exiting an angle-cleaved GRIN will refract at the glass–air interface, changing its propagation direction. Here, we introduce an offset between the SMF and GRIN (Figure 3b), which changes the direction of the beam propagating in the GRIN. This is similar to placing the SMF off-axis in front of a classical lens, making the beam leave the lens under an angle with respect to propagation axis. A larger offset results in a larger change in the beam direction. We design this offset to make the two effects (due to the offset and angle-cleave) cancel each other out. In a preliminary report,^32^ we demonstrated that this technique can reduce the level of back-reflection, but not to the desired value below −60 dB. We explain later what further modifications were needed to achieve this. Splicing of such a structure that includes offset-spliced SMF and angle-cleaved HCF as demonstrated here has not been demonstrated so far.

## Design

**Figure 4:**
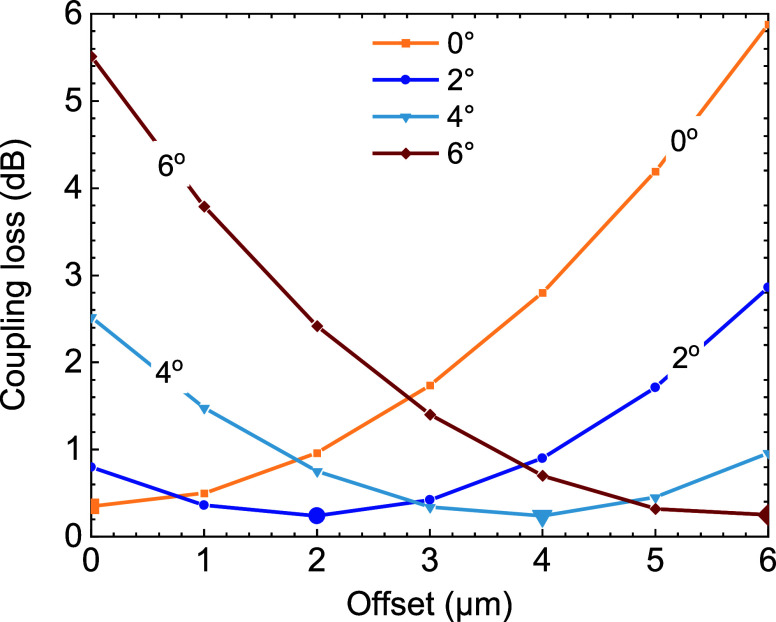
Relationship between coupling loss and offset of light propagated through GRINs with different cleave angles to an HCF.

**Figure 5:**
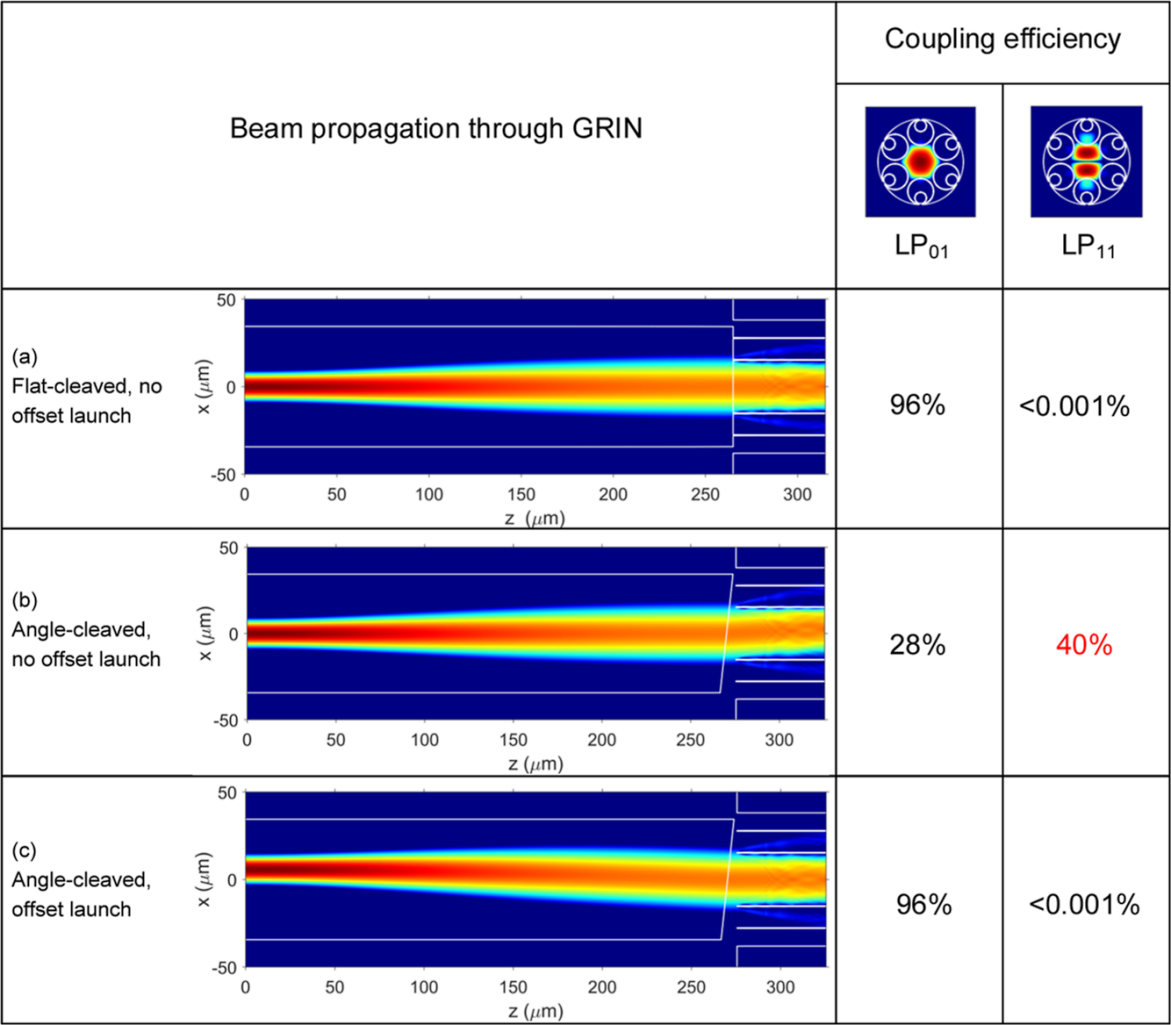
Simulated beam propagated through the GRIN and HCF together with the coupling efficiency into the HCF’s LP_01_ mode and parasitic cross-coupling into the LP_11_ mode using an example in which 6° cleave angle is considered. Mode field profiles of the HCF’s LP_01_ and LP_11_ are also shown. Configurations shown: (a) GRIN with zero cleave angle and launch with zero offset; (b) GRIN with angle cleave of 6° with zero offset; and (c) GRIN with angle cleave of 6° and optimized offset of 5.7 μm (c). It can be appreciated that the beam entering HCF propagates along the *z*-axis for (a,c) (as schematically shown in Figure 3b), while it propagates under an angle in (b), as schematically shown in Figure 3a. This gives rise to coupling into the LP_11_ mode and reduces the coupling into the LP_01_ mode.

We simulated propagation through the SMF–GRIN–HCF, considering a range of SMF–GRIN offsets (0–6 μm) and a range of GRIN cleave angles (0–6°) using beam propagation method implemented in MATLAB with BeamLab software.^33^ The simulated coupling loss between the input beam and the fundamental mode of the HCF (LP_01_) as well as parasitic cross-coupling into the HCF’s higher order modes (e.g., LP_11_) is calculated as.
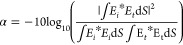
1where *E*_*i*_ is the transverse electric field component of the beam propagated at the HCF input, and *E*_t_ is the transverse electric field component of the considered HCF mode (e.g., LP_01_, LP_11_, etc.). The calculated insertion loss (which we define as the coupling loss into the HCF’s fundamental mode LP_01_) is shown in Figure 4. The input light was a Gaussian beam with a 10 μm waist, representing the mode field profile of a standard SMF operating at 1550 nm. The considered HCF was a 6-ring nested antiresonant nodeless fiber (NANF)^34^ with a core size of 30 μm, which corresponds to parameters of the HCF used in experiments. The refractive index profile of the GRIN corresponded to the GRIN we manufactured in-house and used in the experiments. It has a parabolic refractive index profile with a core size of 50 μm. Its length was set to quarter pitch (265 μm) that generates a collimated beam at the GRIN output. From Figure 4, we see that for each cleave angle (0, 2, 4, and 6° are shown), there is an offset that minimizes the coupling loss. As the minimum achievable coupling loss does not depend on the cleave angle, Figure 4, this technique effectively breaks the trade-off between the loss and achievable back-reflection as the back-reflection decreases with the cleave angle (as we discuss in detail later). Figure 5 illustrates three scenarios of beam propagation together with the insertion loss and cross-coupling into the first higher order (LP_11_) mode, defined in eq 1. First, as a benchmark, we show a zero angle cleave with zero offset in Figure 5a. As expected, the input beam was enlarged when propagating through the GRIN, matching that of the HCF. The coupling loss into the LP_01_ mode is very low (coupling efficiency of 96%) with negligible unwanted cross-coupling into the LP_11_ (<0.001%), thanks to the perfect symmetry of the simulated input beam, GRIN, and HCF. Figure 5b shows the situation where the GRIN is angle-cleaved at 6° and the offset is zero. This represents a situation previously published.^25^ Here, we see that the coupling efficiency is severely reduced (to 28% in our case) with significant cross-coupling into the LP_11_ mode, as expected for a launch in which the input beam is not colinear with the HCF axis. Introducing an optimized offset of 5.7 μm for the considered cleave angle of 6° (as follows from Figure 4), Figure 5c, the low coupling loss is restored, with the coupling efficiency reaching 96% and the cross-coupling into the LP_11_ being negligible within the simulation error (below −50 dB, <0.001%).

Consequently, coupling loss as well as cross-coupling into LP_11_ are the same for the flat-cleaved, zero-offset case (Figure 5a) and for the optimized offset, angle-cleaved case that achieves <−60 dB back-reflection (Figure 5c).

**Figure 6:**
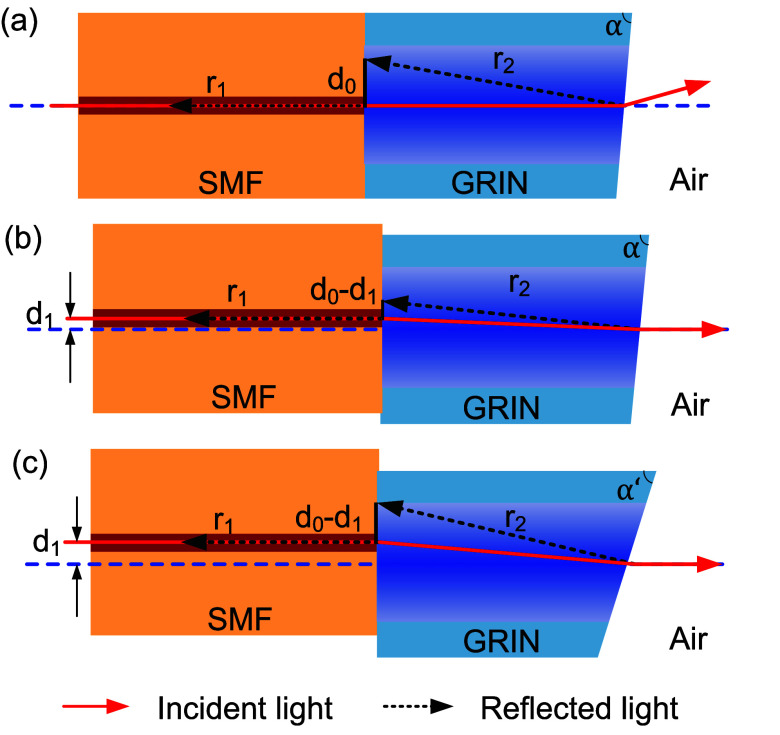
Schematics of the reflections from the interface between SMF and angled GRIN and angled GRIN and air. (a) Configuration with zero offset launch; light back-reflected from GRIN accumulates *d*_0_ offset when re-entering the SMF. (b) By introducing offset launch *d*_1_, back-reflection coupled back into the SMF is increased as back-reflected beam enters SMF with a smaller offset of *d*_0_ – *d*_1_. (c) Both offset and cleave angle need to be increased to achieve the same back-reflection level as in (a).

So far, we have established what the relationship is between the GRIN cleave angle and the offset to obtain the lowest coupling loss. Now, we need to establish how the cleavage angle influences the back-reflection level. As we sketched in Figure 6, the back-reflection depends on three parameters, which includes the cleave angle, offset *d*_1_, and back-reflection *r*_1_ from the SMF–GRIN interface. The direction of the reflection *r*_2_ from the GRIN–air interface depends on the cleave angle, causing the *r*_2_ reflected beam to enter the SMF with an offset *d*_0_. The coupling efficiency of *r*_2_ back into the SMF depends on the offset *d*_0_ – *d*_1_, Figure 6b,c, which, for no-offset (*d*_1_ = 0, Figure 6a), reduces to *d*_0_. Consequently, a larger cleave angle is required for a larger offset *d*_1_, which reduces (*d*_0_ – *d*_1_) and thus increases the level of back-reflection. Thus, to achieve both low coupling loss and low back-reflection, we need to increase simultaneously the cleave angle and offset with respect to the no-offset situation, as shown in Figure 6c.

**Figure 7:**
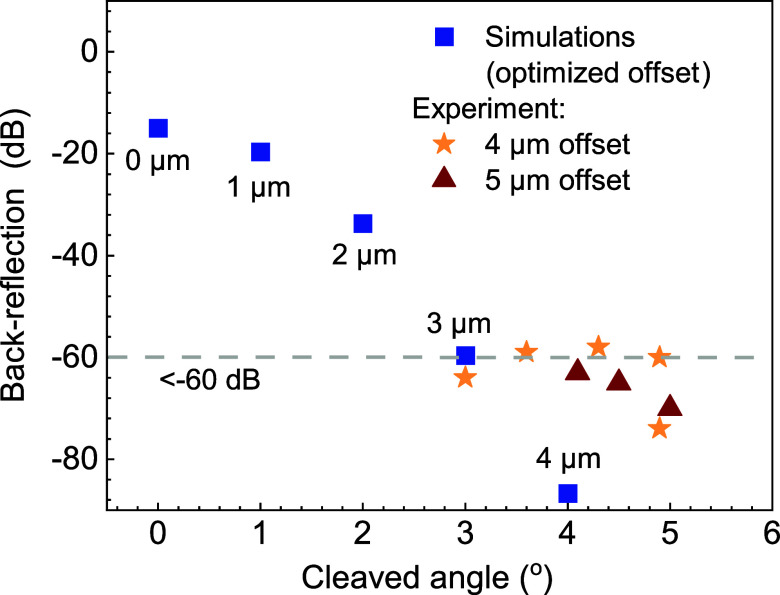
Simulated and measured back-reflection from angle-cleaved GRINs with SMF–GRIN offset. Simulations: offset (given next to the data points) optimized for minimum coupling loss. Experiment: offset of 4 μm (yellow stars) and 5 μm (red triangles).

We simulated the back-reflection into the SMF to find the minimum cleave angle required to achieve a back-reflection below −60 dB. The blue squares in Figure 7 show the result for an optimized offset obtained from the data presented in Figure 4. It indicates that the cleave angle must be over 3°.

## Experiment and Discussion

In the previous analysis, we have not discussed back-reflection at the SMF–GRIN interface *r*_1_ (Figure 6). Based on our analysis below, we believe this back-reflection was not negligible in the published zero-offset SMF–GRIN splices (Figure 6a). This required our attention as this contribution could be potentially even larger for the offset splice (Figure 6c), depending on the refractive index difference between the SMF core and GRIN at the point where it is spliced to the SMF.

**Figure 8:**
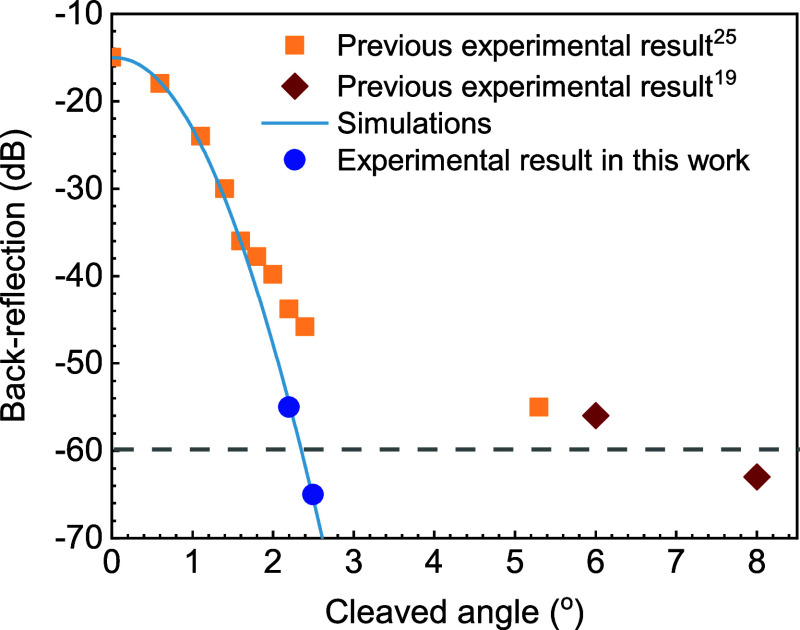
Back-reflection from zero-offset angle-cleaved SMF–GRIN: simulations (blue line), data from literature (red^19^ and yellow^25^ squares), and results achieved experimentally here (blue circles).

First, we simulated the back-reflected power from the GRIN–air interface with various angles using BeamLab using backward propagating light (Figure 8, blue solid line). The results show that a back-reflection below −60 dB requires a cleave angle as low as 2.4°. However, although published experimental results^23,25^ (Figure 8, yellow and red squares) show good agreement with the simulations up to 1.5°, there is appreciable discrepancy for larger angles, requiring cleave angles as large as 8° to achieve −60 dB of back-reflection.

We thus first optimized the splicing recipe to reduce any potential contribution from *r*_1_. A significantly longer splice time with reduced power allows the dopants in the SMF and GRIN to diffuse, making the refractive index variation at the SMF–GRIN interface smoothened and, thus, the associated Fresnel reflection *r*_1_ reduced. Using this procedure, the measured back-reflection level (Figure 8, blue circles) agrees with the simulations (Figure 8, blue line). This suggests that the previously reported results were limited by the non-negligible Fresnel back-reflection *r*_1_ at the SMF–GRIN interface.

**Figure 9:**
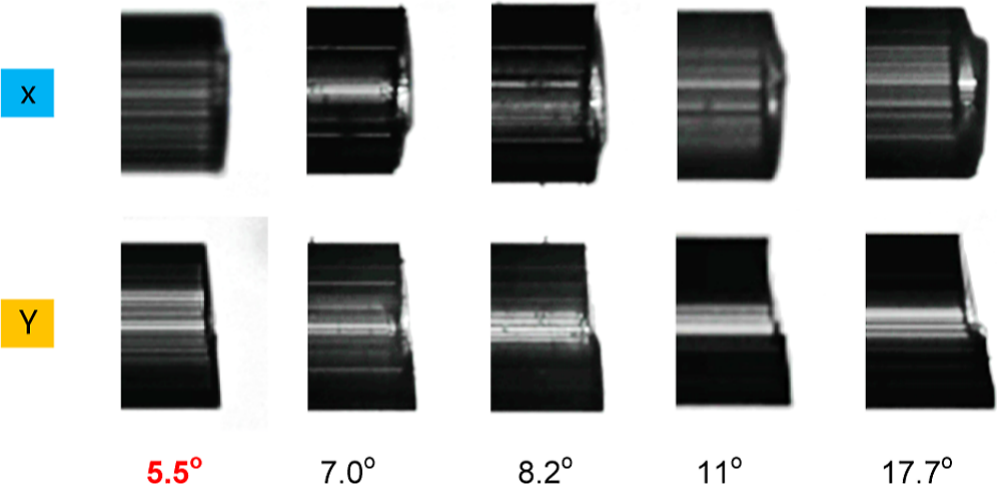
Photographs of angle-cleaved HCFs. The angle value given here is evaluated by the fusion splicer software.

In the next step, we angle-cleaved the HCF using a CT-105 cleaver from Fujikura with an in-house attached rotation stage to enable angle-cleaving. Subsequently, we spliced it to the angle-cleaved GRIN. For angles up to 6°, the cleaved surface was relatively even. However, for larger angles, the cleaved surface showed increasing levels of unevenness, Figure 9. Such an uneven surface is expected to lead to an increase in the insertion loss during fusion splicing, reducing the mechanical strength or even producing a connection that is not airtight. It is worth mentioning that an alternative method of end-face polishing that would give better surface quality would be challenging to use, as the debris from the polishing would penetrate into the HCF microstructure.

From simulations (Figure 7), our technique requires a cleave angle > 3° to achieve −60 dB back-reflection, while cleave angles below 6° resulted in an end-facet surface of sufficient quality. Thus, we targeted cleave angle of 4–5°, which should be large enough to achieve back-reflection below −60 dB, but small enough to get an even-surface angle-cleaved HCF. For this cleave angle range, our simulations predicted the optimum offset to be 4.0–4.7 μm. We tested two offset values of 4 and 5 μm, paying attention to applying the offset in the same direction as that of the GRIN angled cleave. To apply the offset, we first cladding-aligned the two fibers in the splicer and then moved the fiber-holding stage in the splicer by the desired offset. Subsequently, we measured the back-reflections of the fabricated angled GRINs offset-spliced with SMF. The results are shown together with the previously discussed simulations in Figure 7 as yellow stars (4 μm offset) and red triangles (5 μm offset). Based on the results, we chose to use an offset of 5 μm, as all the prepared SMF–GRINs had back-reflection below −60 dB, Figure 7. Although the obtained back-reflection below −60 dB is sufficiently low, we would expect even lower levels from the simulations, as shown in Figure 7. We believe this discrepancy may be due to the angle cleave and offset directions not being perfectly aligned.

**Figure 10:**
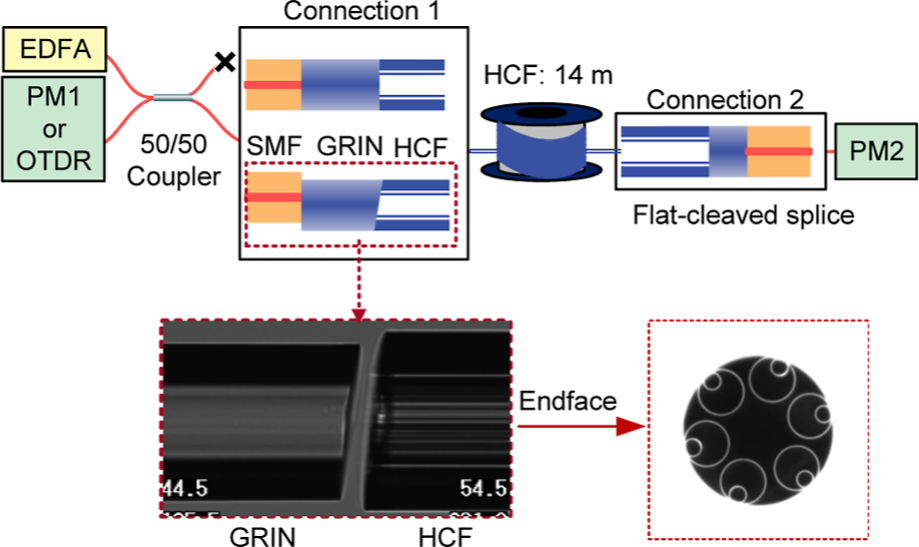
Setup for insertion loss and back-reflection measurements. EDFA: erbium-doped fiber amplifier; PM: power meter. When back-reflection is measured by the OTDR, the EDFA and PMs were disconnected, and Connection 2 was removed. Inset: aligned angle-cleaved GRIN with angle-cleaved HCF prior to fusion splicing.

The used HCF was 14 m long, had six nested ring NANF geometry with a core diameter of 30 μm and transmission loss of 0.6 dB/km, and was designed for operation in the first antiresonant window. The loss of the 14 m long sample used here is 0.008 dB, which can be neglected. An end-face image is shown as an inset in Figure 10. As HCFs are multimoded, certain care must be taken to measure the coupling loss into the HCF’s fundamental mode correctly, especially when dealing with relatively short lengths of HCF where the higher order modes do not get completely attenuated.^35^ We used the measurement procedure described in ref (19). In this procedure, both ends of the HCF are connected identically, and the splice loss of each of these two connections is then estimated as 1/2 of the total insertion loss. First, we spliced both ends with flat-cleaved SMF–GRINs, obtaining a total insertion loss of 1.2 dB. As both ends have the same configuration, we estimate the coupling loss at each end as 1.2/2 = 0.6 dB. Subsequently, we cut one of the ends and replaced it with the studied offset-spliced angle-cleaved SMF–GRIN.

We offset-spliced the SMF–GRIN cleaved at an angle of 4.5°, which showed back-reflection of −65 dB. Subsequently, we aligned it in the splicer with an HCF cleaved with an angle close to 4.5°. The measurement setup is shown in Figure 10, where the studied interface is referred to as “Connection 1”. We used amplified spontaneous emission (ASE) from an erbium-doped fiber amplifier (EDFA) as the unpolarized, broadband light source and connected power meter 1 (PM1) to measure the back-reflection from the offset-spliced SMF–GRIN and power meter 2 (PM2) to measure the power at the output. The measured loss was 1.2 dB, which indicates that the coupling loss from offset-angle-cleaved SMF–GRIN to HCF was about 0.6 dB, close to that of the flat-cleaved connection. This is consistent with our simulations shown in Figure 4, which predict that coupling loss of the offset-spliced angle-cleaved connection can be similar to zero-offset-spliced flat-cleaved connection.

After splicing, the loss unfortunately increased by 0.6 dB. For flat-cleaved splices, we have observed that the fusion splicing process contributes as little as 0.1 dB of additional loss. Thus, we believe that the achieved additional loss due to the splicing can be reduced by optimizing the splice recipe, obtaining a better match between the cleave angles of the GRIN and SMF, or by improving the cleave angle uniformity. We also believe that the entire process could be implemented using commercially available cleavers (such as CT-116 from Fujikura) and splices (such as ARC Master FSM-100M+ from Fujikura).

**Figure 11:**
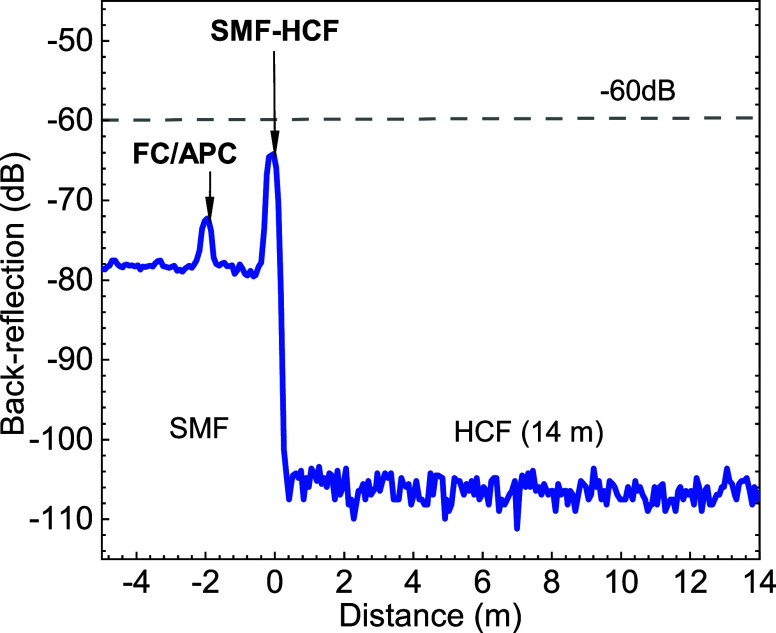
Measured OTDR trace showing back-reflection in the offset-spliced SMF–GRIN angle-spliced to the HCF.

Subsequently, we cut off the flat-cleaved connection and characterized the back-reflection using two techniques. First is based on direct back-reflection power measurement (using PM1, Figure 10), giving a value of −64 dB. Subsequently, we used an optical time domain reflectometer (OTDR, LOR-200 from Luciol Instruments S. A., Switzerland, pulse width of 2 ns). The measured OTDR trace is shown in Figure 11.

In the OTDR trace, Figure 11, we see that the backscattering level from the SMF and HCF differs by about 27 dB, consistently with previous reports.^30^ Importantly for us, the level of back-reflection from the SMF–HCF interface is −64 dB, in good agreement with the direct back-reflection power measurement. It is also consistent with the back-reflection value of −65 dB measured at the used SMF–GRIN offset-spliced, angle-cleaved component (before aligning and splicing the HCF at its end).

The obtained spliced connection thus has −64 dB back-reflection with 1.2 dB loss, which could potentially reach 0.6 dB when reducing the splice-induced additional loss.

## Conclusions

We demonstrated a novel splice-connection method for integrating HCFs into SMF-based systems that can achieve simultaneously low back-reflection and low loss. We optimized it via simulations and subsequently achieved <−60 dB back-reflection, while achieving insertion loss at 1 dB level. Although this is already acceptable in a wide range of applications, further insertion loss reduction is possible via optimization of the GRIN, HCF angle-cleaving, and splicing recipe with expected loss potentially reaching as low as 0.25 dB. Such level is consistent with splicing of two dissimilar solid glass-core fibers such as SMF and a dispersion–compensation fiber. Thus, our results pave the way to seamless integration of HCFs into SMF system using well-established and widely accepted method of fusion splicing that shows good long-term stability, mechanical stability, operation over wide temperature range, and so on. The results obtained here should be transferable to other geometries of antiresonant HCFs. HCFs have many unique properties in comparison with the SMF, but many components or subsystems are nowadays available only with SMF. Thus, connection demonstrated here is expected to enable designing fiber optic systems that would combine the best of both technologies.

## Supporting Information Available

Data underpinning the research presented are accessible through the University of Southampton research repository (DOI: 10.5258/SOTON/D3154). For the purpose of open access, the author has applied a creative commons attribution (CC BY) license to any author accepted manuscript version arising (PDF)
