# Supplementary material for: Splicing Hollow-Core Fiber with Standard Glass-Core Fiber with Ultralow Back-Reflection and Low Coupling Loss
Source: ACS Photonics. 2024 Jul 29;11(8):3288–95. doi: 10.1021/acsphotonics.4c00677 (PMC11342409; doi:10.1021/acsphotonics.4c00677)
Supplement: Supplementary file 1 — ph4c00677_si_001.pdf [file ph4c00677_si_001.pdf]

Splicing hollow core fiber with standard glass-core fiber with ultralow back-reflection and low coupling loss

Bo Shi, Cong Zhang, Thomas Kelly, Xuhao Wei, Meng Ding, Meng Huang, Songnian Fu， Francesco Poletti, and Radan Slavík

Supporting Information for Publication

The data underpinning the research presented are accessible through the University of Southampton research repository (DOI: 10.5258/SOTON/D3154). For the purpose of open access, the author has applied a creative commons attribution (CC BY) license to any author accepted manuscript version arising.
